# The effects of fentanyl and dexmedetomidine on cardiorespiratory activity: implications for morbidity and mortality associated with adulterated fentanyl products

**DOI:** 10.3389/fphar.2026.1885956

**Published:** 2026-09-17

**Authors:** Mark A. Smith, Emma M. Thomas, Stephanie G. Hockett, Kirsten J. Folger

**Affiliations:** 1 Department of Psychology, Davidson College, Davidson, NC, United States; 2 Program in Neuroscience, Davidson College, Davidson, NC, United States; 3 Department of Biology, Davidson College, Davidson, NC, United States

**Keywords:** alpha-2 adrenergic agonist, cardiorespiratory toxicity, fentanyl, medetomidine, opioid overdose, respiratory depression

## Abstract

**Introduction:**

A recent driver of opioid-related overdose deaths has been the adulteration of illicitly manufactured fentanyl with alpha-2 (α_2_)-adrenergic agonists; however, little research has examined their toxicity when combined with fentanyl. The purpose of this study was to examine the effects of fentanyl and the α_2_-adrenergic agonist, dexmedetomidine, on cardiorespiratory measures that may contribute to the toxicity of these drug combinations (i.e., respiratory rate, heart rate, oxygen (O_2_) saturation, and pulse distention).

**Methods:**

Male and female rats were administered fentanyl (0.01–0.1 mg/kg) and dexmedetomidine (0.003–0.01 mg/kg) intravenously, and pulse oximetry was used to measure respiratory and cardiovascular activity.

**Results:**

When administered alone, both drugs decreased respiratory rate and heart rate relative to baseline, albeit with different time courses. Fentanyl, but not dexmedetomidine, decreased O_2_ saturation. Dexmedetomidine enhanced the suppression of respiratory rate and heart rate produced by fentanyl in a time-dependent manner. The effects of the drug combination on O_2_ saturation and pulse distention were less consistent. Although not a primary endpoint, mortality occurred in 3 of 16 rats after the high-dose drug combination, suggesting that increases in lethality can be observed in the absence of explicit pharmacological synergy.

**Discussion:**

These data indicate that dexmedetomidine exacerbates the cardiorespiratory effects of fentanyl in a manner that may contribute to the morbidity and mortality associated with α_2_-adrenergic adulteration of illicitly manufactured fentanyl.

## Introduction

Opioid overdose remains a major U.S. public health crisis driven largely by illicitly manufactured fentanyl and complicated by polysubstance exposure (Centers for Disease Control and Prevention, 2023a; Centers for Disease Control and Prevention, 2023b; Jones et al., 2018; Gladden et al., 2016; O'Donnell et al., 2017). Alpha-2 (α_2_)-adrenergic receptor agonists, including xylazine and medetomidine, are increasingly encountered as fentanyl adulterants and can produce sedation, bradycardia, altered vascular tone, and physiological depression (Kariisa et al., 2023; Drug Enforcement Administration, 2023; Centers for Disease Control and Prevention, 2023c; Gipson and Strickland, 2024; Hoffman, 2023; Office of National Drug Control Policy, 2023; Huo and Perrone, 2026; Centers for Disease Control and Prevention, 2026; Sinclair, 2003; Murrell and Hellebrekers, 2005; Giovannitti et al., 2015). Medetomidine has been identified in drug material, overdose patients, and drug-checking samples, with reports of profound sedation, bradycardia, incomplete naloxone response, and withdrawal (Centers for Disease Control and Prevention, 2026; Sisco and Appley, 2023; Schwarz et al., 2024; Sibley et al., 2025; Nham et al., 2025; Huo et al., 2025).

Fentanyl and α_2_-adrenergic agonists act through distinct receptor systems but produce overlapping physiological depression. Fentanyl causes μ-opioid receptor-mediated respiratory depression and hypoxemia, whereas α_2_-adrenergic agonists reduce sympathetic outflow and produce sedation and bradycardia (Boom et al., 2012; Saunders and Levitt, 2020; Palkovic et al., 2020; Furdui et al., 2023; Sinclair, 2003; Murrell and Hellebrekers, 2005; Giovannitti et al., 2015). Their combination may therefore alter respiratory, cardiac, oxygenation, and peripheral pulse-oximetry measures in dissociable ways and may complicate pharmacological reversal (Dahan et al., 2024; Choi et al., 2024; Datta et al., 2025).

The present study characterized the acute effects of intravenous fentanyl and dexmedetomidine, the pharmacologically active enantiomer of medetomidine, on respiratory rate, heart rate, oxygen saturation, and pulse distention in awake, freely moving rats. Dexmedetomidine was used to isolate the α_2_-adrenergic component most likely to contribute to medetomidine-associated toxicity. The study was designed to characterize acute cardiorespiratory toxicity and endpoint-specific drug interactions, not to identify their complete mechanistic basis.

## Materials and methods

### Subjects and drugs

Sixteen Long-Evans rats (8 male, 8 female; 56 days old at arrival; Charles River Laboratories, Raleigh, NC, United States) were housed individually under controlled temperature, humidity, and a 12-h light/dark cycle. Rats were maintained at approximately 90% of sex- and age-adjusted free-feeding bodyweight to stabilize bodyweight and body composition across repeated testing, with water and enrichment available continuously. Procedures were approved by the Davidson College Animal Care and Use Committee (Protocol 7-25-78), and the manuscript was prepared in accordance with Animal Research: Reporting of *In Vivo* Experiments (ARRIVE) guidelines, as applicable. Fentanyl citrate and dexmedetomidine hydrochloride (Cayman Chemical, Ann Arbor, MI, United States) were dissolved in sterile 0.9% saline and administered by manual tail-vein injection (1 mL/kg); no indwelling catheters were used. Conditions included saline, fentanyl (0.01, 0.03, 0.1 mg/kg), dexmedetomidine (0.003, 0.01 mg/kg), and all six dose combinations. Doses were selected from behaviorally and physiologically active ranges reported in rats and extended from minimally effective single-drug conditions to doses affecting at least two cardiorespiratory measures (Hu et al., 1992; Zalischi et al., 2026; Marchette et al., 2023; Gaulden et al., 2021; Garrity et al., 2015; Kitajima et al., 2022). They were intended to examine graded pharmacological interactions rather than reproduce a specific human exposure ratio.

### Cardiorespiratory monitoring and procedure

Respiratory rate, heart rate, oxygen saturation, and pulse distention were recorded with a MouseOx Plus® system (Starr Life Sciences Corp., Oakmont, PA, United States) in awake rats. After collar and injection habituation, rats completed a 10-min baseline period, received one intravenous injection, and were monitored for 90 min. Conditions were tested in a pseudorandomized repeated-measures order, generally twice weekly with at least 3 days between tests. When multiple rats were monitored, the system cycled among animals, yielding measurements from each rat at intervals of approximately 4 min; thus, very rapid post-injection effects may not have been captured. Additional housing, habituation, monitoring, injection, and test-order details are provided in the Supplementary Methods.

### Data analysis

Three rats (2 male, 1 female) died within 10 min after receiving 0.1 mg/kg fentanyl plus 0.01 mg/kg dexmedetomidine. Mortality was retained as a descriptive outcome for the full cohort of 16, whereas inferential analyses and visualizations used the 13 rats that completed the repeated-measures design. Data were averaged into one 10-min baseline bin and nine post-injection bins. Twenty-six of 6,240 observations (0.4%) were missing and were imputed as described in the Supplementary Methods. Sensitivity analyses excluding imputed observations produced the same pattern of statistically significant effects and did not alter the primary conclusions.

Linear mixed-effects models were fit by maximum likelihood with subject as a random intercept. Sex was modeled as a between-subjects factor and dose or condition and time as within-subjects factors; Type III tests used Satterthwaite approximations. Estimated marginal means were used for planned comparisons. Dunnett-adjusted tests compared each post-injection bin with baseline for single-drug analyses and compared the high-dose combination with each single drug within time bins. Trough analyses used minimum values across the session and Tukey-adjusted comparisons among dexmedetomidine levels within fentanyl dose. Statistical significance was set at α = 0.05. Complete model results and sex-stratified analyses are provided in Supplementary Tables S1–S3 and Supplementary Figures S1–S3.

## Results

### Single-drug effects of fentanyl and dexmedetomidine on cardiorespiratory activity

Fentanyl produced time-dependent decreases in respiratory rate, heart rate, and oxygen saturation, with more limited effects on pulse distention (dose × time interactions, p ≤ 0.0147; Figure 1; Supplementary Table S1). The 0.1 mg/kg dose decreased respiratory rate for 30 min, heart rate for 20 min, oxygen saturation for 30 min, and pulse distention from 30 to 60 min. Dexmedetomidine produced sustained decreases in respiratory rate and heart rate. The 0.01 mg/kg dose decreased respiratory rate from 20 to 90 min and heart rate throughout the session, whereas 0.003 mg/kg decreased heart rate at selected time points. Dexmedetomidine increased pulse distention over the middle of the session and produced a small, delayed increase in oxygen saturation at 80 and 90 min (4.5 and 5.5 percentage points, respectively).

**FIGURE 1:**
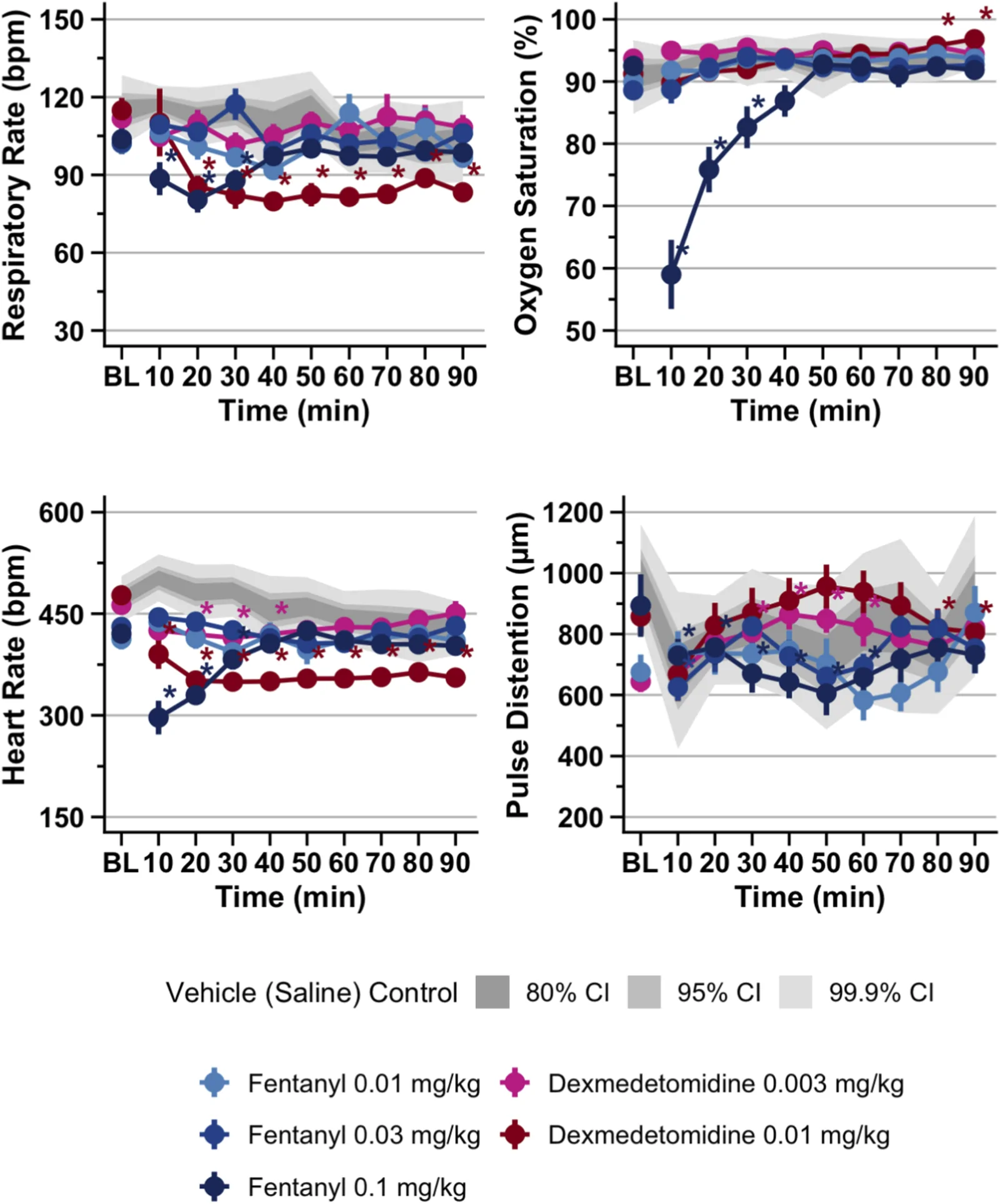
Effects of intravenous doses of fentanyl and dexmedetomidine on cardiorespiratory measures. All panels depict the mean ± standard error of the mean (SEM) for 0.003 mg/kg dexmedetomidine, 0.01 mg/kg dexmedetomidine, 0.01 mg/kg fentanyl, 0.03 mg/kg fentanyl, and 0.1 mg/kg fentanyl over a 90-min session. The x-axes show 10-min bins immediately prior to drug administration (baseline: BL) and following drug injection. The y-axes show respiratory rate in breaths/min (bpm; top left panel), oxygen saturation as a percentage (O_2_ saturation; top right panel), heart rate in beats/min (bpm; bottom left panel), and pulse distention in micrometers (μm; bottom right panel). Shaded regions indicate 80%, 95%, and 99.9% confidence intervals (CIs) for each time point generated during a vehicle (saline) control session. Asterisks (*) indicate values significantly different from baseline; color indicates the comparison for the relevant dose/drug condition.

### Drug combinations of fentanyl and dexmedetomidine on cardiorespiratory activity

Trough analyses showed that fentanyl and dexmedetomidine each decreased respiratory rate and heart rate (all main effects, p < 0.0001; Figure 2; Supplementary Table S2), and their combined effects were greater than either drug’s effects when administered alone. A fentanyl × dexmedetomidine interaction was observed for heart rate (p = 0.0114), reflecting greater bradycardia when 0.01 mg/kg dexmedetomidine was combined with 0.03 mg/kg fentanyl. Fentanyl decreased oxygen saturation and pulse distention, whereas dexmedetomidine did not significantly alter either endpoint in the trough analysis.

**FIGURE 2:**
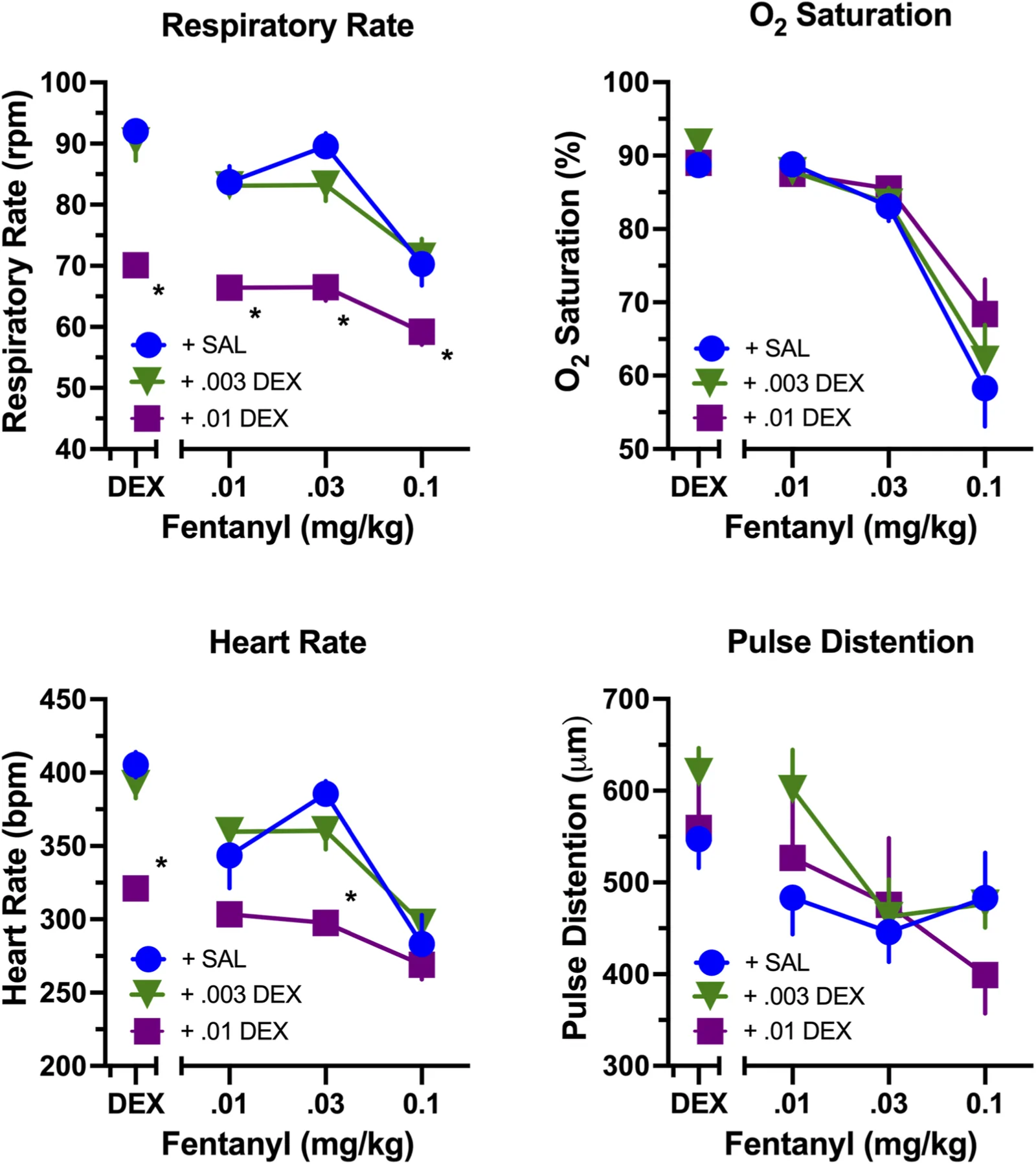
Interactions between fentanyl and dexmedetomidine on trough (minimum) values obtained across 90-min test sessions. All panels depict the mean ± SEM of the minimum value obtained from each rat, regardless of when that value occurred during the 90-min session. The x-axes show fentanyl dose, including the dexmedetomidine-alone condition, in combination with saline, 0.003 mg/kg dexmedetomidine, or 0.01 mg/kg dexmedetomidine. The y-axes show respiratory rate in respirations/min (rpm; top left panel), oxygen saturation as a percentage (O_2_ saturation; top right panel), heart rate in beats/min (bpm; bottom left panel), and pulse distention in micrometers (μm; bottom right panel). Asterisks (*) indicate values significantly different from fentanyl administered alone.

### Cardiorespiratory effects of a high-dose fentanyl and dexmedetomidine combination

Three rats died within 10 min after the high-dose combination (0.1 mg/kg fentanyl + 0.01 mg/kg dexmedetomidine; Supplementary Table S4). Reliable post-injection MouseOx data were unavailable for these animals. Among the 13 completers, the combination produced time-dependent effects on all four endpoints (condition × time interactions, p ≤ 0.0004; Figure 3; Supplementary Table S3).

**FIGURE 3:**
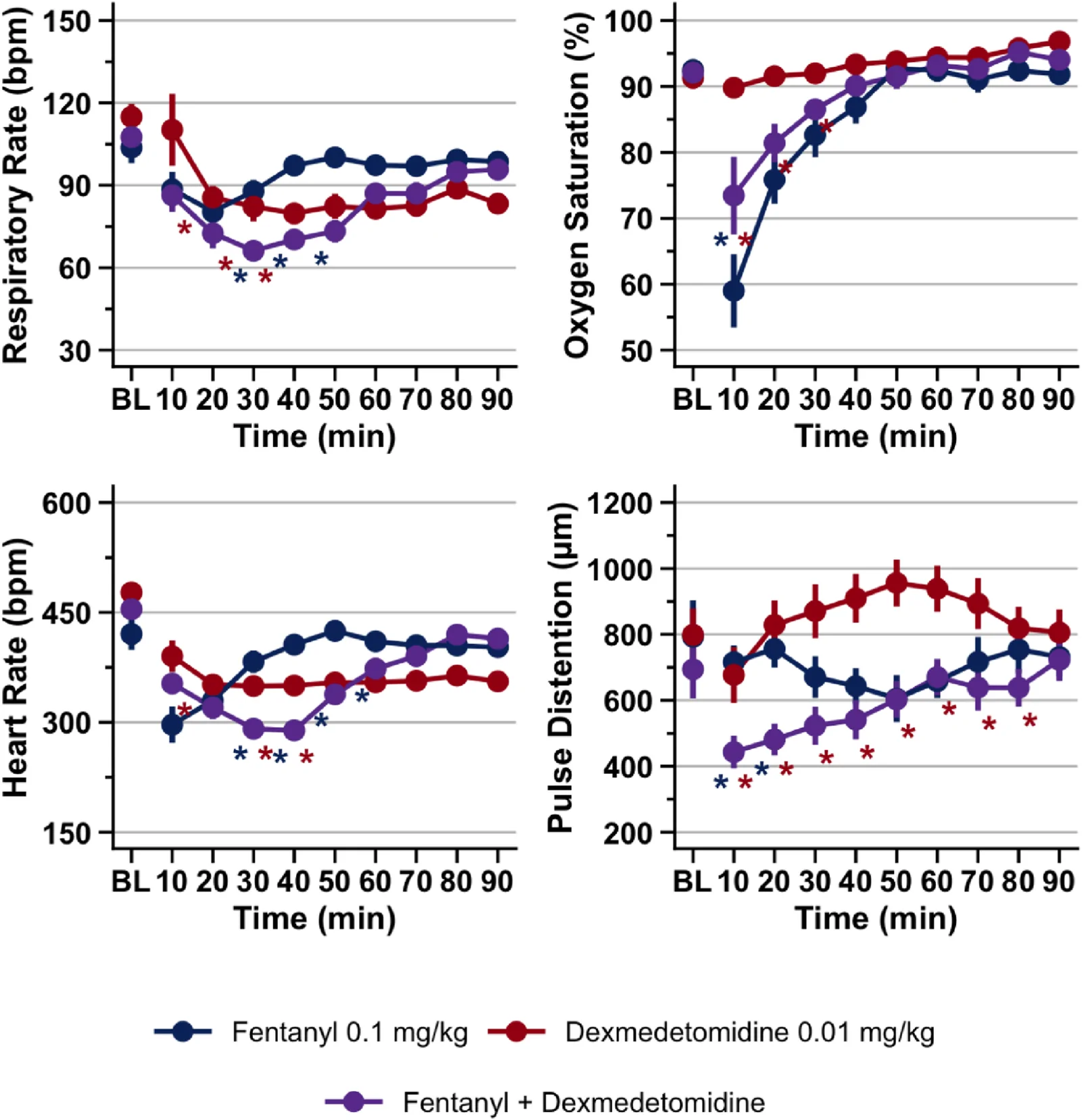
Effects of a high-dose combination of fentanyl and dexmedetomidine on cardiorespiratory measures. All panels depict the mean ± SEM for 0.1 mg/kg fentanyl, 0.01 mg/kg dexmedetomidine, and their combination over a 90-min session. The x-axes show 10-min bins immediately prior to drug administration (baseline: BL) and following drug injection. The y-axes show respiratory rate in breaths/min (bpm; top left panel), oxygen saturation as a percentage (O_2_ saturation; top right panel), heart rate in beats/min (bpm; bottom left panel), and pulse distention in micrometers (μm; bottom right panel). Asterisks (*) indicate values of the drug combination significantly different from fentanyl and/or dexmedetomidine alone; color indicates the comparison for the relevant drug condition.

Respiratory-rate suppression was greater than fentanyl alone at 30–50 min and greater than dexmedetomidine alone at 10–30 min. Heart-rate suppression was greater than fentanyl alone at 30–60 min and greater than dexmedetomidine alone at 10, 30, and 40 min, although heart rate was higher than with dexmedetomidine alone late in the session. The combination differed from fentanyl alone in oxygen saturation only during the first 10 min and was lower than dexmedetomidine alone during the first 30 min. Pulse distention was lower than fentanyl alone during the first 20 min and lower than dexmedetomidine alone through 80 min.

## Discussion

Fentanyl and dexmedetomidine both impaired respiratory and cardiac function but with distinct time courses, and dexmedetomidine enhanced or prolonged fentanyl-induced decreases in respiratory rate and heart rate under selected conditions. Oxygen saturation and pulse distention showed different patterns, and the highest-dose combination was associated with mortality in 3 of 16 rats. Thus, the toxicological profile of the combination was endpoint specific and was not captured by any single physiological measure.

The respiratory and heart-rate findings are consistent with overlapping μ-opioid and α_2_-adrenergic depression. Fentanyl suppresses pontomedullary respiratory networks and can increase parasympathetic cardiac control, whereas medetomidine reduces sympathetic outflow and can produce reflex and vagally mediated bradycardia (Saunders and Levitt, 2020; Palkovic et al., 2020; Sinclair, 2003; Murrell and Hellebrekers, 2005; Pattinson, 2008; Laubie et al., 1977; Griffioen et al., 2004; Savola, 1989; Shimizu et al., 2012; Chen and Ashburn, 2015). The differing time courses observed here are consistent with prior fentanyl-medetomidine and fentanyl-xylazine studies showing that α_2_-adrenergic agonists can intensify physiological suppression without uniformly producing synergistic interactions across endpoints (Hu et al., 1992; Zalischi et al., 2026; Puzovic et al., 2025; Demery et al., 2025).

Oxygen saturation did not parallel respiratory rate. Fentanyl-induced hypoxemia may reflect respiratory depression and, at high exposure, rigidity that impairs respiratory mechanics (Saunders and Levitt, 2020; Palkovic et al., 2020; Pattinson, 2008; Haouzi and Tubbs, 2022). In contrast, dexmedetomidine generally has modest direct respiratory effects when administered alone (Sinclair, 2003; Venn et al., 2000). The late dexmedetomidine-associated increase was small, delayed, and of uncertain physiological significance; reduced metabolic demand, altered ventilation-perfusion relationships, compensatory responses, or pulse-oximetry signal effects are possible explanations. Pulse distention likewise should be interpreted only as an exploratory surrogate of pulsatile signal amplitude, not a direct measure of blood flow, vascular resistance, or vasoconstriction.

The present design cannot formally classify the interaction as additive, synergistic, or subadditive because it did not use fixed-ratio dose-addition or isobolographic methods. Prior work demonstrated synergistic fentanyl-xylazine lethality, whereas fixed-ratio cardiorespiratory studies found largely additive or subadditive interactions (Puzovic et al., 2025; Smith et al., 2023). Mortality in the present study therefore remains descriptive but toxicologically important. Excluding the three animals that died also likely underestimates the severity of the high-dose condition. Both sexes were included, but sex-related effects were inconsistent, and the study was not powered to define sex-specific mechanisms, consistent with variable sex effects reported previously (Zalischi et al., 2026; Marchette et al., 2023; Puzovic et al., 2025; Smith et al., 2023).

Several limitations remain. Dexmedetomidine was used rather than racemic medetomidine, and the other enantiomer could alter apparent potency, pharmacokinetics, or toxicity. The approximately 4-min MouseOx sampling interval may have missed rapid post-injection effects. Although stable baselines and the pseudorandomized schedule make substantial carryover or tolerance unlikely, tolerance was not tested directly. Restraint and injection transiently altered heart rate and pulse distention after vehicle administration. Finally, metabolic rate, arterial blood gases, direct vascular measures, and plasma or brain drug concentrations were not obtained; mechanistic and pharmacokinetic explanations therefore remain hypotheses for future study.

## Conclusion

Dexmedetomidine modifies fentanyl-induced cardiorespiratory depression in a measure-specific manner and can contribute to severe toxicity at high-dose combinations. These findings support treating emerging α_2_-adrenergic adulterants as active contributors to fentanyl-involved overdose rather than passive contaminants.

## Data Availability

All original data from this study are openly available at Mendeley Data: https://data.mendeley.com/datasets/6jwbywy5jp/1.
