# Supplementary material for: The effects of fentanyl and dexmedetomidine on cardiorespiratory activity: implications for morbidity and mortality associated with adulterated fentanyl products
Source: Front Pharmacol. 2026 Sep 17;17:1885956. doi: 10.3389/fphar.2026.1885956 (PMC13627786; doi:10.3389/fphar.2026.1885956)

Supplementary Figure 3: High Dose Combination Faceted by Sex (refer to Figure 3)

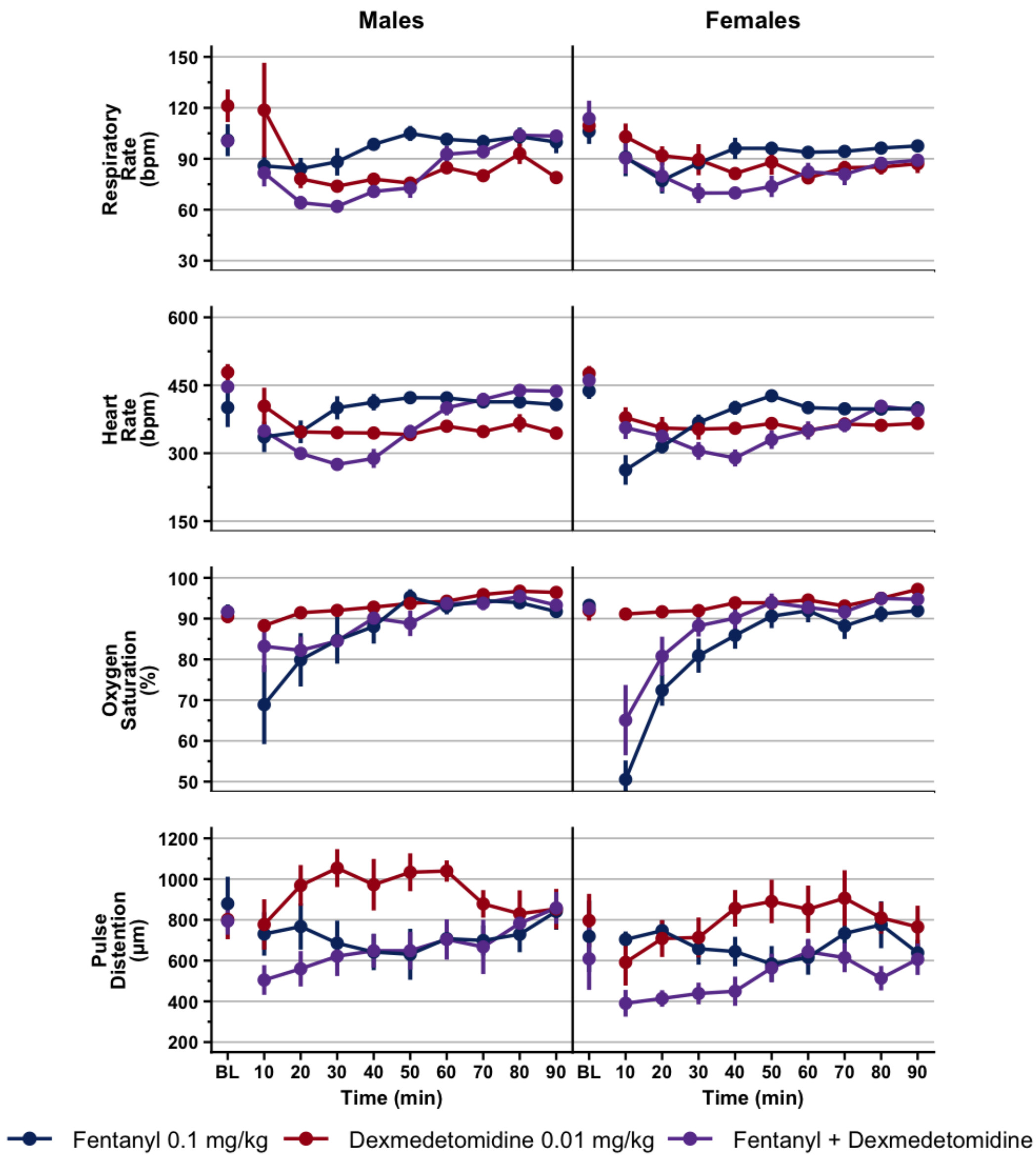

Supplement: Supplementary file 3 [file Supplementaryfile3.pdf]
