# Supplementary material for: The effects of fentanyl and dexmedetomidine on cardiorespiratory activity: implications for morbidity and mortality associated with adulterated fentanyl products
Source: Front Pharmacol. 2026 Sep 17;17:1885956. doi: 10.3389/fphar.2026.1885956 (PMC13627786; doi:10.3389/fphar.2026.1885956)
Supplement: Supplementary file 4 [file Supplementaryfile4.pdf]

Supplementary Methods

Animals were housed individually in transparent polycarbonate cages in a temperature- and humidity-controlled colony room on a 12-hr light/dark cycle (lights on at 0500); testing occurred during the light phase. Rats had continuous access to water and enrichment materials, including bedding, gnaw sticks, and plastic tubs.

The MouseOx Plus® system used pulse-oximetry collars connected by cable to a multiplexer. When multiple rats were tested, the system cycled sequentially among animals, collecting 5 s of valid oxygen-saturation data per rat before advancing; if no valid reading was obtained within 30 s, it advanced to the next rat. With eight rats, each animal was sampled at minimum intervals of approximately 4 min. Collars were promptly replaced if dislodged.

Before testing, rats wore manufacturer-supplied training collars for 1-2 hr on 3-4 days (5-6 hr total). Each rat also underwent one restraint-tube session with a saline tail-vein injection. All rats completed habituation. For each test, rats were moved to the testing room 30 min before the session, placed in individual monitoring cages, fitted with collars, and monitored for 10 min. Rats were then briefly restrained, injected manually into a tail vein, returned immediately to the monitoring cage, and observed for 90 min.

Drug conditions were pseudorandomized so that no more than two ascending or two descending doses of either drug were tested consecutively whether administered alone or in combination. Sessions were generally conducted twice weekly with at least 3 days between tests, and no rat received more than three acute exposures to either drug within any 2-week period.

Recorded values were averaged within 10-min bins. Missing interior bins were imputed by linear interpolation from the adjacent bins. For a missing first post-injection bin, the 20-min value was carried backward; for a missing 90-min bin, the 80-min value was carried forward. Sensitivity models excluded all imputed observations.

For the time-course models, fixed effects included sex, dose or drug condition, time, and their interactions. Planned estimated-marginal-mean comparisons averaged across sex while retaining sex in the model. For each single-drug dose, one Dunnett family comprised the nine post-injection bins compared with baseline. For the high-dose comparison, fentanyl alone and dexmedetomidine alone were compared with the combination within each post-baseline bin. Trough analyses used each rat's minimum value regardless of time and included sex, fentanyl dose, dexmedetomidine dose, and all interactions; Tukey-adjusted comparisons were performed among dexmedetomidine levels within fentanyl dose. Kenward-Roger confidence intervals were obtained for estimated marginal means, and model convergence and residual distributions were inspected visually.
