# Supplementary material for: The effects of fentanyl and dexmedetomidine on cardiorespiratory activity: implications for morbidity and mortality associated with adulterated fentanyl products
Source: Front Pharmacol. 2026 Sep 17;17:1885956. doi: 10.3389/fphar.2026.1885956 (PMC13627786; doi:10.3389/fphar.2026.1885956)
Supplement: Supplementary file 5 [file Supplementaryfile5.pdf]

Supplementary Table 1: Type III Mixed-Model ANOVA Results for Each Drug Alone

Analysis	Effect	df(N)	df(D)	F	p
**Fent RR**	Sex (S)	1	13	2.38	0.1472
	Dose (D)	2	377	17.30	< 0.0001
	Time (T)	9	377	1.54	0.1314
	S x D	2	377	0.58	0.5610
	S x T	9	377	1.11	0.3522
	D x T	18	377	3.26	< 0.0001
	S x D x T	18	377	1.05	0.4062
**Fent HR**	Sex (S)	1	13	0.92	0.3548
	Dose (D)	2	377	25.52	< 0.0001
	Time (T)	9	377	1.95	0.0445
	S x D	2	377	1.34	0.2622
	S x T	9	377	1.59	0.1174
	D x T	18	377	7.24	< 0.0001
	S x D x T	18	377	1.18	0.2747
**Fent O2**	Sex (S)	1	13	0.76	0.3984
	Dose (D)	2	377	50.67	< 0.0001
	Time (T)	9	377	17.76	< 0.0001
	S x D	2	377	9.18	0.0001
	S x T	9	377	2.14	0.0257
	D x T	18	377	12.87	< 0.0001
	S x D x T	18	377	0.94	0.5284
**Fent PD**	Sex (S)	1	13	4.13	0.0630
	Dose (D)	2	377	1.97	0.1411
	Time (T)	9	377	2.83	0.0031
	S x D	2	377	3.62	0.0279
	S x T	9	377	0.79	0.6302
	D x T	18	377	1.90	0.0147
	S x D x T	18	377	1.10	0.3523
**Dex RR**	Sex (S)	1	13	0.22	0.6435
	Dose (D)	1	247	89.10	< 0.0001
	Time (T)	9	247	4.77	< 0.0001
	S x D	1	247	0.11	0.7423
	S x T	9	247	1.31	0.2342
	D x T	9	247	4.09	< 0.0001
	S x D x T	9	247	1.40	0.1894
**Dex HR**	Sex (S)	1	13	0.83	0.3796
	Dose (D)	1	247	182.96	< 0.0001
	Time (T)	9	247	13.22	< 0.0001
	S x D	1	247	1.74	0.1879
	S x T	9	247	0.35	0.9559
	D x T	9	247	4.63	< 0.0001
	S x D x T	9	247	0.81	0.6103
**Dex O2**	Sex (S)	1	13.305	0.28	0.6052
	Dose (D)	1	243.365	12.44	0.0005
	Time (T)	9	243.507	3.88	0.0001
	S x D	1	243.365	0.00	0.9524
	S x T	9	243.507	0.50	0.8762
	D x T	9	243.299	3.84	0.0001
	S x D x T	9	243.299	0.72	0.6864
**Dex PD**	Sex (S)	1	13.009	2.08	0.1727
	Dose (D)	1	246.016	16.83	< 0.0001
	Time (T)	9	246.015	5.45	< 0.0001
	S x D	1	246.016	0.52	0.4704
	S x T	9	246.015	2.73	0.0048
	D x T	9	246.015	1.08	0.3747
	S x D x T	9	246.015	0.73	0.6832

Fent: Fentanyl
Dex: Dexmedetomidine
RR: Respiratory Rate
HR: Heart Rate
O2: Oxygen Saturation
PD: Pulse Distention
