# Supplementary material for: The effects of fentanyl and dexmedetomidine on cardiorespiratory activity: implications for morbidity and mortality associated with adulterated fentanyl products
Source: Front Pharmacol. 2026 Sep 17;17:1885956. doi: 10.3389/fphar.2026.1885956 (PMC13627786; doi:10.3389/fphar.2026.1885956)
Supplement: Supplementary file 6 [file Supplementaryfile6.pdf]

**Supplementary Table 2: Type III Mixed-Model ANOVA Results for Minimum Values in Combination Tests**

| Analysis | Effect | df(N) | df(D) | F | p |
|---|---|---|---|---|---|
| **MIN RR** | Sex | 1 | 13.075 | 0.00 | 0.9881 |
| | Fent | 2 | 103.196 | 30.53 | < 0.0001 |
| | Dex | 2 | 103.196 | 58.67 | < 0.0001 |
| | Sex × Fent | 2 | 103.196 | 0.50 | 0.6061 |
| | Sex × Dex | 2 | 103.196 | 2.52 | 0.0856 |
| | Fent × Dex | 4 | 103.194 | 2.03 | 0.0952 |
| | Sex × Fent × Dex | 4 | 103.194 | 0.95 | 0.4374 |
| **MIN HR** | Sex | 1 | 13.075 | 0.01 | 0.9144 |
| | Fent | 2 | 103.169 | 31.54 | < 0.0001 |
| | Dex | 2 | 103.169 | 21.42 | < 0.0001 |
| | Sex × Fent | 2 | 103.169 | 0.95 | 0.3891 |
| | Sex × Dex | 2 | 103.169 | 1.21 | 0.3012 |
| | Fent × Dex | 4 | 103.168 | 3.42 | 0.0114 |
| | Sex × Fent × Dex | 4 | 103.168 | 3.30 | 0.0138 |
| **MIN O2** | Sex | 1 | 116 | 4.10 | 0.0452 |
| | Fent | 2 | 116 | 65.21 | < 0.0001 |
| | Dex | 2 | 116 | 1.40 | 0.2507 |
| | Sex × Fent | 2 | 116 | 4.56 | 0.0124 |
| | Sex × Dex | 2 | 116 | 0.09 | 0.9100 |
| | Fent × Dex | 4 | 116 | 1.05 | 0.3866 |
| | Sex × Fent × Dex | 4 | 116 | 0.47 | 0.7596 |
| **MIN PD** | Sex | 1 | 13.066 | 10.48 | 0.0064 |
| | Fent | 2 | 103.134 | 5.87 | 0.0039 |
| | Dex | 2 | 103.134 | 1.21 | 0.3031 |
| | Sex × Fent | 2 | 103.134 | 6.23 | 0.0028 |
| | Sex × Dex | 2 | 103.134 | 11.66 | < 0.0001 |
| | Fent × Dex | 4 | 103.133 | 1.74 | 0.1465 |
| | Sex × Fent × Dex | 4 | 103.133 | 2.04 | 0.0947 |

**MIN:** Minimum
**Fent:** Fentanyl
**Dex:** Dexmedetomidine
**RR:** Respiratory Rate
**HR:** Heart Rate
**O2:** Oxygen Saturation
**PD:** Pulse Distention
