# Supplementary material for: The effects of fentanyl and dexmedetomidine on cardiorespiratory activity: implications for morbidity and mortality associated with adulterated fentanyl products
Source: Front Pharmacol. 2026 Sep 17;17:1885956. doi: 10.3389/fphar.2026.1885956 (PMC13627786; doi:10.3389/fphar.2026.1885956)
Supplement: Supplementary file 7 [file Supplementaryfile7.pdf]

Supplementary Table 3: Type III Mixed-Model ANOVA Results for 0.1 Fentanyl and 0.01 Dexmedetomidine

Analysis	Effect	df(N)	df(D)	F	p
**Combo RR**	Sex	1	13	0.13	0.7253
	Dose	2	377	16.92	< 0.0001
	Time	9	377	13.59	< 0.0001
	Sex × Dose	2	377	0.78	0.4581
	Sex × Time	9	377	1.47	0.1585
	Dose × Time	18	377	4.30	< 0.0001
	Sex × Dose × Time	18	377	1.34	0.1618
**Combo HR**	Sex	1	13.02	1.04	0.3267
	Dose	2	376.04	12.87	< 0.0001
	Time	9	376.04	28.39	< 0.0001
	Sex × Dose	2	376.04	2.49	0.0844
	Sex × Time	9	376.04	1.39	0.1916
	Dose × Time	18	376.04	11.19	< 0.0001
	Sex × Dose × Time	18	376.04	1.43	0.1153
**Combo O2**	Sex	1	13	1.81	0.2018
	Dose	2	377	40.16	< 0.0001
	Time	9	377	35.12	< 0.0001
	Sex × Dose	2	377	4.20	0.0157
	Sex × Time	9	377	2.92	0.0024
	Dose × Time	18	377	6.28	< 0.0001
	Sex × Dose × Time	18	377	1.25	0.2167
**Combo PD**	Sex	1	13	2.06	0.1752
	Dose	2	377	64.80	< 0.0001
	Time	9	377	2.55	0.0076
	Sex × Dose	2	377	3.07	0.0477
	Sex × Time	9	377	0.85	0.5688
	Dose × Time	18	377	2.59	0.0004
	Sex × Dose × Time	18	377	0.87	0.6145

Combo: Combination of 0.1 Fentanyl and 0.01 Dexmedetomidine
RR: Respiratory Rate
HR: Heart Rate
O2: Oxygen Saturation
PD: Pulse Distention
