# Supplementary material for: The effects of fentanyl and dexmedetomidine on cardiorespiratory activity: implications for morbidity and mortality associated with adulterated fentanyl products
Source: Front Pharmacol. 2026 Sep 17;17:1885956. doi: 10.3389/fphar.2026.1885956 (PMC13627786; doi:10.3389/fphar.2026.1885956)
Supplement: Supplementary file 8 [file Supplementaryfile8.pdf]

Supplementary Table 4: Descriptive Analysis of Mortality Data

Outcome	Value
Rats entering study	16
Deaths after high-dose combination	3
Sex of rats that died	2 male, 1 female
Approximate time to death	within 10 min
Rats completing full repeated-measures design	13
Inferential analysis cohort	13 completers
Mortality analysis	descriptive only
